# 4-(4-Bromo­phen­yl)-2-oxo-1,2,5,6-tetra­hydro­benzo[*h*]quinoline-3-carbonitrile

**DOI:** 10.1107/S1600536811033885

**Published:** 2011-08-27

**Authors:** Abdullah M. Asiri, Hassan M. Faidallah, Abdulrahman O. Al-Youbi, Khalid A. Alamry, Seik Weng Ng

**Affiliations:** a Chemistry Department, Faculty of Science, King Abdulaziz University, PO Box 80203 Jeddah, Saudi Arabia; b Department of Chemistry, University of Malaya, 50603 Kuala Lumpur, Malaysia

## Abstract

In the mol­ecule of the title compound, C_20_H_13_BrN_2_O, the tetra­hydro­benzo[*h*]quinoline fused-ring system is buckled owing to the ethyl­ene –CH_2_CH_2_– fragment, the benzene ring and the pyridine ring being twisted by 17.7 (1)°. The 4-substituted aromatic ring is bent away from the pyridine ring by 82.3 (1)° in order to avoid crowding the cyanide substituent. Two mol­ecules are linked by a pair of N—H⋯O hydrogen bonds to form a centrosymmetric dimer.

## Related literature

For background to the anti­cancer properties of this class of compounds, see: Rostom *et al.* (2011 ▶).
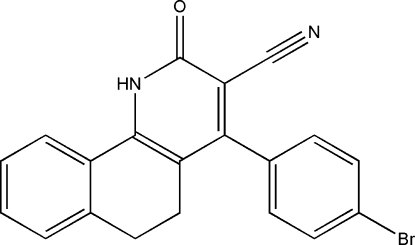


## Experimental

### 

#### Crystal data

C_20_H_13_BrN_2_O
*M* *_r_* = 377.23
Monoclinic, 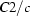

*a* = 22.6906 (5) Å
*b* = 8.5060 (2) Å
*c* = 17.6112 (5) Å
β = 106.498 (3)°
*V* = 3259.13 (14) Å^3^
*Z* = 8
Cu *K*α radiation
μ = 3.50 mm^−1^
*T* = 100 K
0.30 × 0.25 × 0.20 mm

#### Data collection

Agilent SuperNova Dual diffractometer with Atlas detector
Absorption correction: multi-scan (*CrysAlis PRO*; Agilent, 2010 ▶) *T*_min_ = 0.420, *T*_max_ = 0.541
6063 measured reflections
3244 independent reflections
3132 reflections with *I* > 2σ(*I*)
*R*_int_ = 0.022

#### Refinement

*R*[*F* ^2^ > 2σ(*F* ^2^)] = 0.032
*wR*(*F* ^2^) = 0.094
*S* = 1.06
3244 reflections
221 parameters
H atoms treated by a mixture of independent and constrained refinement
Δρ_max_ = 0.56 e Å^−3^
Δρ_min_ = −0.57 e Å^−3^

### 

Data collection: *CrysAlis PRO* (Agilent, 2010 ▶); cell refinement: *CrysAlis PRO*; data reduction: *CrysAlis PRO*; program(s) used to solve structure: *SHELXS97* (Sheldrick, 2008 ▶); program(s) used to refine structure: *SHELXL97* (Sheldrick, 2008 ▶); molecular graphics: *X-SEED* (Barbour, 2001 ▶); software used to prepare material for publication: *publCIF* (Westrip, 2010 ▶).

## Supplementary Material

Crystal structure: contains datablock(s) global, I. DOI: 10.1107/S1600536811033885/xu5291sup1.cif

Structure factors: contains datablock(s) I. DOI: 10.1107/S1600536811033885/xu5291Isup2.hkl

Supplementary material file. DOI: 10.1107/S1600536811033885/xu5291Isup3.cml

Additional supplementary materials:  crystallographic information; 3D view; checkCIF report

## Figures and Tables

**Table 1:** Hydrogen-bond geometry (Å, °)

*D*—H⋯*A*	*D*—H	H⋯*A*	*D*⋯*A*	*D*—H⋯*A*
N1—H1⋯O1^i^	0.86 (3)	1.96 (3)	2.807 (2)	172 (3)

Symmetry code: (i) 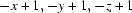.

**Fig. 1.**
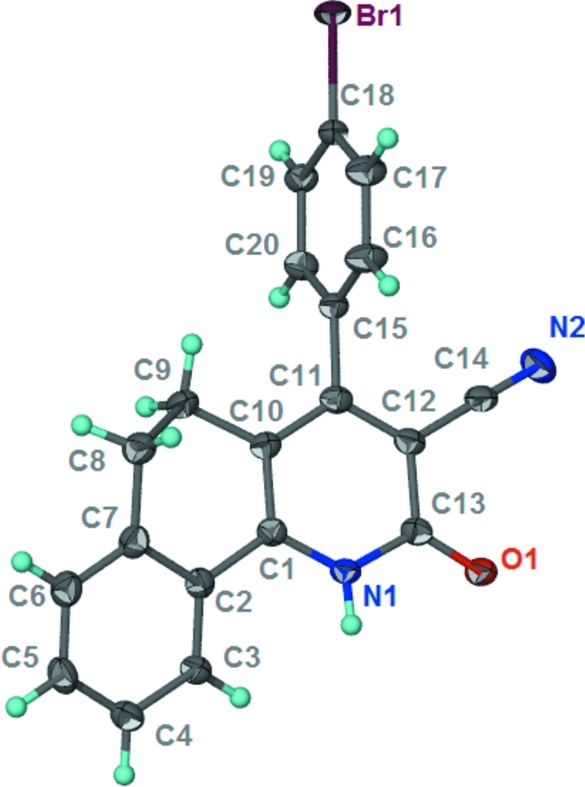
Thermal ellipsoid plot (Barbour, 2001) of C20H13BrN2O at the 70% probability level; hydrogen atoms are drawn as spheres of arbitrary radius.

## supplementary crystallographic information

### Comment

The compound (Scheme I) belongs to a series of cyano-pyridinones that have been evaluated for their anticancer properties (Rostom *et al.*, 2011). The tetrahydrobenzo[*h*]quinoline fused-ring system is buckled owing to the ethylene –CH_2_CH_2_– fragment, the benzene ring and the pyridine ring being twisted by 17.7 (1)°. The 4-subsituted aromatic ring is bent away from the pyridine ring by 83.2 (1) ° in order to avoid crowding the cyanide substituent (Fig. 1). Two molecules are linked by an N—H···O hydrogen bonds to form a centrosymmetric dimer (Table 1).

### Experimental

A mixture of *p*-bromobenzaldehyde (1.85 g, 10 mmol), 1-tetralone (1.46 g, 10 mmol), ethyl cyanoacetate (1.1 g, 10 mmol) and ammonium acetate (6.2 g, 80 mmol) in absolute ethanol (50 ml) was refluxed for 6 h. The reaction mixture was allowed to cool, and the orange precipitate that formed was filtered, washed with water, dried and recrystallized from ethanol; m.p. >630 K.

### Refinement

Carbon- and nitrogen-bound H atoms were placed in calculated positions [C—H 0.95 to 0.99 Å, *U*_iso_(H) = 1.2*U*_eq_(C)] and were included in the refinement in the riding model approximation.

The amino H atom was located in a difference Fourier map and was freely refined.

### Crystal data

**Table:**

C_20_H_13_BrN_2_O	*F*(000) = 1520
*M**_r_* = 377.23	*D*_x_ = 1.538 Mg m^−^^3^
Monoclinic, *C*2/*c*	Cu *K*α radiation, λ = 1.54184 Å
Hall symbol: -C 2yc	Cell parameters from 4349 reflections
*a* = 22.6906 (5) Å	θ = 4.2–74.3°
*b* = 8.5060 (2) Å	µ = 3.50 mm^−^^1^
*c* = 17.6112 (5) Å	*T* = 100 K
β = 106.498 (3)°	Octahedron, yellow
*V* = 3259.13 (14) Å^3^	0.30 × 0.25 × 0.20 mm
*Z* = 8	

### Data collection

**Table:**

Agilent SuperNova Dual diffractometer with Atlas detector	3244 independent reflections
Radiation source: SuperNova (Cu) X-ray Source	3132 reflections with *I* > 2σ(*I*)
mirror	*R*_int_ = 0.022
Detector resolution: 10.4041 pixels mm^-1^	θ_max_ = 74.4°, θ_min_ = 5.2°
ω scans	*h* = −27→27
Absorption correction: multi-scan (*CrysAlis PRO*; Agilent, 2010)	*k* = −7→10
*T*_min_ = 0.420, *T*_max_ = 0.541	*l* = −14→21
6063 measured reflections	

### Refinement

**Table:**

Refinement on *F*^2^	Primary atom site location: structure-invariant direct methods
Least-squares matrix: full	Secondary atom site location: difference Fourier map
*R*[*F*^2^ > 2σ(*F*^2^)] = 0.032	Hydrogen site location: inferred from neighbouring sites
*wR*(*F*^2^) = 0.094	H atoms treated by a mixture of independent and constrained refinement
*S* = 1.06	*w* = 1/[σ^2^(*F*_o_^2^) + (0.0634*P*)^2^ + 3.4873*P*] where *P* = (*F*_o_^2^ + 2*F*_c_^2^)/3
3244 reflections	(Δ/σ)_max_ = 0.001
221 parameters	Δρ_max_ = 0.56 e Å^−^^3^
0 restraints	Δρ_min_ = −0.57 e Å^−^^3^

### Fractional atomic coordinates and isotropic or equivalent isotropic displacement parameters (Å^2^)

**Table:**

	*x*	*y*	*z*	*U*_iso_*/*U*_eq_	
Br1	0.918537 (8)	−0.05660 (2)	0.742895 (12)	0.02179 (11)	
O1	0.57369 (7)	0.53089 (18)	0.51145 (10)	0.0241 (3)	
N1	0.53722 (7)	0.3095 (2)	0.55547 (10)	0.0172 (3)	
N2	0.73458 (8)	0.5190 (3)	0.57289 (12)	0.0264 (4)	
C1	0.54467 (9)	0.1682 (2)	0.59371 (11)	0.0167 (4)	
C2	0.48982 (9)	0.0824 (2)	0.60012 (12)	0.0175 (4)	
C3	0.43270 (9)	0.1549 (2)	0.58744 (12)	0.0195 (4)	
H3	0.4284	0.2638	0.5751	0.023*	
C4	0.38190 (10)	0.0686 (2)	0.59274 (14)	0.0223 (4)	
H4	0.3432	0.1187	0.5848	0.027*	
C5	0.38810 (10)	−0.0913 (3)	0.60971 (13)	0.0244 (4)	
H5	0.3533	−0.1510	0.6123	0.029*	
C6	0.44492 (10)	−0.1637 (3)	0.62285 (13)	0.0240 (4)	
H6	0.4487	−0.2728	0.6349	0.029*	
C7	0.49638 (10)	−0.0790 (2)	0.61870 (12)	0.0203 (4)	
C8	0.55804 (9)	−0.1564 (3)	0.63177 (14)	0.0253 (4)	
H8A	0.5629	−0.1912	0.5803	0.030*	
H8B	0.5602	−0.2505	0.6655	0.030*	
C9	0.61001 (10)	−0.0444 (2)	0.67123 (14)	0.0240 (5)	
H9A	0.6096	−0.0241	0.7264	0.029*	
H9B	0.6499	−0.0932	0.6728	0.029*	
C10	0.60314 (9)	0.1088 (2)	0.62645 (11)	0.0181 (4)	
C11	0.65406 (9)	0.1960 (2)	0.61906 (11)	0.0179 (4)	
C12	0.64485 (9)	0.3399 (2)	0.58053 (11)	0.0177 (4)	
C13	0.58428 (9)	0.4025 (2)	0.54653 (12)	0.0178 (4)	
C14	0.69504 (10)	0.4366 (2)	0.57531 (13)	0.0193 (4)	
C15	0.71808 (8)	0.1347 (2)	0.65091 (11)	0.0176 (4)	
C16	0.74530 (10)	0.0579 (3)	0.59968 (13)	0.0252 (5)	
H16	0.7228	0.0448	0.5456	0.030*	
C17	0.80490 (10)	0.0000 (3)	0.62658 (12)	0.0251 (4)	
H17	0.8232	−0.0532	0.5916	0.030*	
C18	0.83693 (9)	0.0216 (2)	0.70531 (12)	0.0182 (4)	
C19	0.81110 (9)	0.0985 (2)	0.75721 (12)	0.0194 (4)	
H19	0.8340	0.1127	0.8110	0.023*	
C20	0.75125 (9)	0.1550 (2)	0.72992 (12)	0.0200 (4)	
H20	0.7330	0.2073	0.7652	0.024*	
H1	0.5015 (13)	0.349 (3)	0.5343 (15)	0.026 (7)*	

### Atomic displacement parameters (Å^2^)

**Table:**

	*U*^11^	*U*^22^	*U*^33^	*U*^12^	*U*^13^	*U*^23^
Br1	0.01372 (15)	0.02727 (16)	0.02247 (15)	0.00536 (7)	0.00206 (10)	0.00254 (7)
O1	0.0149 (7)	0.0195 (7)	0.0366 (9)	0.0000 (6)	0.0050 (6)	0.0078 (6)
N1	0.0119 (7)	0.0173 (8)	0.0211 (8)	−0.0001 (6)	0.0026 (6)	0.0008 (6)
N2	0.0188 (9)	0.0326 (10)	0.0289 (10)	−0.0042 (8)	0.0084 (8)	0.0001 (8)
C1	0.0157 (9)	0.0182 (9)	0.0154 (8)	−0.0006 (7)	0.0032 (7)	−0.0010 (7)
C2	0.0176 (9)	0.0195 (9)	0.0147 (9)	−0.0015 (8)	0.0034 (7)	0.0000 (7)
C3	0.0160 (9)	0.0202 (9)	0.0217 (9)	−0.0007 (8)	0.0043 (7)	0.0011 (8)
C4	0.0161 (10)	0.0266 (11)	0.0242 (11)	−0.0010 (8)	0.0054 (8)	0.0009 (8)
C5	0.0209 (10)	0.0280 (11)	0.0241 (10)	−0.0076 (9)	0.0063 (8)	0.0018 (9)
C6	0.0237 (10)	0.0210 (10)	0.0257 (10)	−0.0040 (8)	0.0046 (8)	0.0032 (8)
C7	0.0217 (10)	0.0198 (10)	0.0184 (10)	−0.0014 (8)	0.0039 (8)	0.0011 (7)
C8	0.0214 (10)	0.0193 (10)	0.0340 (11)	0.0006 (8)	0.0062 (9)	0.0048 (9)
C9	0.0172 (10)	0.0245 (11)	0.0278 (11)	0.0024 (8)	0.0023 (9)	0.0085 (8)
C10	0.0153 (9)	0.0193 (10)	0.0186 (9)	0.0012 (8)	0.0028 (7)	0.0019 (8)
C11	0.0155 (9)	0.0210 (9)	0.0162 (8)	0.0011 (8)	0.0028 (7)	−0.0020 (7)
C12	0.0133 (8)	0.0214 (9)	0.0180 (9)	0.0001 (7)	0.0037 (7)	−0.0006 (7)
C13	0.0148 (9)	0.0187 (9)	0.0192 (9)	−0.0006 (8)	0.0037 (7)	−0.0014 (8)
C14	0.0147 (10)	0.0237 (11)	0.0187 (10)	0.0035 (7)	0.0036 (8)	0.0009 (7)
C15	0.0133 (8)	0.0186 (9)	0.0193 (9)	0.0003 (7)	0.0024 (7)	0.0027 (7)
C16	0.0190 (10)	0.0354 (13)	0.0176 (10)	0.0066 (8)	−0.0008 (8)	−0.0028 (8)
C17	0.0216 (10)	0.0333 (12)	0.0193 (10)	0.0065 (9)	0.0039 (8)	−0.0027 (9)
C18	0.0128 (8)	0.0210 (9)	0.0199 (9)	0.0019 (8)	0.0032 (7)	0.0038 (8)
C19	0.0157 (9)	0.0216 (9)	0.0181 (9)	−0.0007 (8)	0.0004 (7)	−0.0005 (8)
C20	0.0177 (9)	0.0225 (10)	0.0195 (9)	0.0018 (8)	0.0050 (8)	−0.0018 (8)

### Geometric parameters (Å, °)

**Table:**

Br1—C18	1.9009 (19)	C8—H8A	0.9900
O1—C13	1.244 (3)	C8—H8B	0.9900
N1—C1	1.365 (3)	C9—C10	1.508 (3)
N1—C13	1.374 (3)	C9—H9A	0.9900
N1—H1	0.86 (3)	C9—H9B	0.9900
N2—C14	1.149 (3)	C10—C11	1.410 (3)
C1—C10	1.383 (3)	C11—C12	1.387 (3)
C1—C2	1.474 (3)	C11—C15	1.494 (3)
C2—C3	1.395 (3)	C12—C14	1.429 (3)
C2—C7	1.410 (3)	C12—C13	1.436 (3)
C3—C4	1.392 (3)	C15—C20	1.392 (3)
C3—H3	0.9500	C15—C16	1.393 (3)
C4—C5	1.391 (3)	C16—C17	1.390 (3)
C4—H4	0.9500	C16—H16	0.9500
C5—C6	1.388 (3)	C17—C18	1.383 (3)
C5—H5	0.9500	C17—H17	0.9500
C6—C7	1.392 (3)	C18—C19	1.382 (3)
C6—H6	0.9500	C19—C20	1.391 (3)
C7—C8	1.504 (3)	C19—H19	0.9500
C8—C9	1.522 (3)	C20—H20	0.9500
			
C1—N1—C13	124.93 (17)	C8—C9—H9B	109.5
C1—N1—H1	121.9 (19)	H9A—C9—H9B	108.1
C13—N1—H1	113.2 (18)	C1—C10—C11	118.91 (18)
N1—C1—C10	119.81 (18)	C1—C10—C9	118.56 (18)
N1—C1—C2	118.98 (17)	C11—C10—C9	122.49 (18)
C10—C1—C2	121.20 (18)	C12—C11—C10	119.75 (18)
C3—C2—C7	119.98 (19)	C12—C11—C15	119.14 (17)
C3—C2—C1	122.38 (18)	C10—C11—C15	121.11 (18)
C7—C2—C1	117.64 (18)	C11—C12—C14	121.83 (18)
C4—C3—C2	120.39 (19)	C11—C12—C13	121.65 (18)
C4—C3—H3	119.8	C14—C12—C13	116.48 (18)
C2—C3—H3	119.8	O1—C13—N1	121.05 (18)
C5—C4—C3	119.7 (2)	O1—C13—C12	124.01 (18)
C5—C4—H4	120.2	N1—C13—C12	114.94 (18)
C3—C4—H4	120.2	N2—C14—C12	177.1 (2)
C6—C5—C4	120.2 (2)	C20—C15—C16	119.48 (18)
C6—C5—H5	119.9	C20—C15—C11	121.61 (18)
C4—C5—H5	119.9	C16—C15—C11	118.89 (18)
C5—C6—C7	121.0 (2)	C17—C16—C15	120.9 (2)
C5—C6—H6	119.5	C17—C16—H16	119.6
C7—C6—H6	119.5	C15—C16—H16	119.6
C6—C7—C2	118.8 (2)	C18—C17—C16	118.6 (2)
C6—C7—C8	121.56 (19)	C18—C17—H17	120.7
C2—C7—C8	119.62 (19)	C16—C17—H17	120.7
C7—C8—C9	111.25 (18)	C17—C18—C19	121.67 (18)
C7—C8—H8A	109.4	C17—C18—Br1	119.04 (16)
C9—C8—H8A	109.4	C19—C18—Br1	119.29 (15)
C7—C8—H8B	109.4	C18—C19—C20	119.37 (19)
C9—C8—H8B	109.4	C18—C19—H19	120.3
H8A—C8—H8B	108.0	C20—C19—H19	120.3
C10—C9—C8	110.52 (18)	C15—C20—C19	120.03 (18)
C10—C9—H9A	109.5	C15—C20—H20	120.0
C8—C9—H9A	109.5	C19—C20—H20	120.0
C10—C9—H9B	109.5		
			
C13—N1—C1—C10	−0.5 (3)	C9—C10—C11—C12	−176.75 (19)
C13—N1—C1—C2	178.78 (18)	C1—C10—C11—C15	−177.91 (18)
N1—C1—C2—C3	−16.9 (3)	C9—C10—C11—C15	4.3 (3)
C10—C1—C2—C3	162.37 (19)	C10—C11—C12—C14	176.52 (19)
N1—C1—C2—C7	162.20 (18)	C15—C11—C12—C14	−4.5 (3)
C10—C1—C2—C7	−18.5 (3)	C10—C11—C12—C13	−0.9 (3)
C7—C2—C3—C4	−0.2 (3)	C15—C11—C12—C13	178.03 (18)
C1—C2—C3—C4	178.85 (19)	C1—N1—C13—O1	−179.27 (19)
C2—C3—C4—C5	−0.9 (3)	C1—N1—C13—C12	0.6 (3)
C3—C4—C5—C6	1.3 (3)	C11—C12—C13—O1	179.99 (19)
C4—C5—C6—C7	−0.6 (3)	C14—C12—C13—O1	2.4 (3)
C5—C6—C7—C2	−0.6 (3)	C11—C12—C13—N1	0.1 (3)
C5—C6—C7—C8	−179.2 (2)	C14—C12—C13—N1	−177.43 (17)
C3—C2—C7—C6	0.9 (3)	C12—C11—C15—C20	96.8 (2)
C1—C2—C7—C6	−178.17 (18)	C10—C11—C15—C20	−84.2 (3)
C3—C2—C7—C8	179.62 (19)	C12—C11—C15—C16	−82.1 (2)
C1—C2—C7—C8	0.5 (3)	C10—C11—C15—C16	96.8 (2)
C6—C7—C8—C9	−146.7 (2)	C20—C15—C16—C17	0.6 (3)
C2—C7—C8—C9	34.7 (3)	C11—C15—C16—C17	179.5 (2)
C7—C8—C9—C10	−51.9 (2)	C15—C16—C17—C18	−0.6 (4)
N1—C1—C10—C11	−0.4 (3)	C16—C17—C18—C19	0.1 (3)
C2—C1—C10—C11	−179.60 (18)	C16—C17—C18—Br1	−179.90 (17)
N1—C1—C10—C9	177.51 (18)	C17—C18—C19—C20	0.4 (3)
C2—C1—C10—C9	−1.7 (3)	Br1—C18—C19—C20	−179.60 (16)
C8—C9—C10—C1	37.1 (3)	C16—C15—C20—C19	0.0 (3)
C8—C9—C10—C11	−145.1 (2)	C11—C15—C20—C19	−178.99 (19)
C1—C10—C11—C12	1.0 (3)	C18—C19—C20—C15	−0.4 (3)

### Hydrogen-bond geometry (Å, °)

**Table:**

*D*—H···*A*	*D*—H	H···*A*	*D*···*A*	*D*—H···*A*
N1—H1···O1^i^	0.86 (3)	1.96 (3)	2.807 (2)	172 (3)

Symmetry codes: (i) −*x*+1, −*y*+1, −*z*+1.
